# Combining organophosphate-treated wall linings and long-lasting insecticidal nets fails to provide additional control over long-lasting insecticidal nets alone against multiple insecticide-resistant *Anopheles gambiae* in Côte d’Ivoire: an experimental hut trial

**DOI:** 10.1186/1475-2875-13-396

**Published:** 2014-10-09

**Authors:** Corine Ngufor, Mouhamadou Chouaïbou, Emile Tchicaya, Benard Loukou, Nestor Kesse, Raphael N’Guessan, Paul Johnson, Benjamin Koudou, Mark Rowland

**Affiliations:** London School of Hygiene and Tropical Medicine, London, WC1E 7HT UK; Centre Suisse de Recherches Scientifiques, Abidjan, Côte d’Ivoire; Robertson Centre for Statistics, University of Glasgow, Glasgow, UK; Pan African Malaria Vector Research Consortium (Pamverc), London, UK

## Abstract

**Background:**

Insecticide-treated wall lining (ITWL) is a new concept in malaria vector control. Some *Anopheles gambiae* populations in West Africa have developed resistance to all the main classes of insecticides. It needs to be demonstrated whether vector control can be improved or resistance managed when non-pyrethroid ITWL is used alone or together with long-lasting insecticidal nets (LLINs) against multiple insecticide-resistant vector populations.

**Methods:**

Two experimental hut trials were carried out as proofs of concept to evaluate pirimiphos methyl (p-methyl)-treated plastic wall lining (WL) and net wall hangings (NWH) used alone and in combination with LLINs against multiple insecticide-resistant *An. gambiae* in Tiassalé, Côte d’Ivoire. Comparison was made to commercial deltamethrin WL and genotypes for *kdr* and *ace-1*^*R*^ resistance were monitored.

**Results:**

The *kdr* and *ace-1*^*R*^ allele frequencies were 0.83 and 0.44, respectively. *Anopheles gambiae* surviving discriminating concentrations of deltamethrin and p-methyl in WHO resistance tests were 57 and 96%, respectively. Mortality of free-flying *An. gambiae* in huts with p-methyl WL and NWH (66 and 50%, respectively) was higher than with pyrethroid WL (32%; P < 0.001). Mortality with LLIN was 63%. Mortality with the combination of LLIN plus p-methyl NWH (61%) or LLIN plus p-methyl WL (73%) did *not* significantly improve upon the LLIN alone or p-methyl WL or NWH alone. Mosquitoes bearing the *ace-1*^*R*^ were more likely to survive exposure to p-methyl WL and NWH. Selection of heterozygote and homozygote *ace-1*^*R*^ or *kdr* genotypes was *not* less likely after exposure to combined LLIN and p-methyl treatments than to single p-methyl treatment. Blood-feeding rates were lower in huts with the pyrethroid LLIN (19%) than with p-methyl WL (72%) or NWH (76%); only LLIN contributed to personal protection.

**Conclusions:**

Combining p-methyl WL or NWH with LLINs provided no improvement in *An. gambiae* control or personal protection over LLIN alone in southern Côte d’Ivoire; neither did the combination manage resistance. Additional resistance mechanisms to *kdr* and *ace-1*^*R*^ probably contributed to the survival of pyrethroid and organophophate-resistant mosquitoes. The study demonstrates the challenge that malaria control programmes will face if resistance to multiple insecticides continues to spread.

## Background

Long-lasting insecticidal nets (LLINs) and indoor residual spraying (IRS) are the most effective and widely used methods for controlling malaria vectors. The recent reductions in malaria morbidity and mortality across Africa has been attributed to a scale-up of these interventions and to better access to diagnostic testing and artemisinin combination therapy (ACT) to treat malaria [1]. Most national malaria control programmes have prioritized universal coverage of LLINs to populations at risk [2]. Campaigns of IRS are particularly appropriate for rapid transmission control. Both approaches require good organization and receptive communities [3]. LLIN effectiveness relies on people regularly sleeping under their nets. IRS is sometimes challenged by the complex organization required and by user-fatigue sometimes associated with recurrent rounds of spraying [3].

The recent development of insecticide-treated wall lining technology [4] offers the prospect of a novel system of insecticide delivery, which is more residual than IRS and requires limited behavioural change. Interior walls can be lined with polymer sheeting (wall lining) or net wall hangings impregnated with insecticide. Using advances in binder technology, these tools can be prepared in a long-lasting format that allows the insecticide to diffuse to the surface in a controlled fashion, making them a long-lasting alternative to IRS. Pyrethroid-treated durable wall lining has been manufactured commercially using this technique and its use on interior wall surfaces has shown potential to improve user compliance and overcome the operational constraints associated with IRS [4, 5]. Durable wall lining has the potential to remain efficacious on home walls for three to four years. However, with the increasing problem of pyrethroid resistance on malaria vector control [6, 7], non-pyrethroid forms of durable lining which can be used against pyrethroid-resistant malaria vectors are urgently needed [7, 8]. Such materials could significantly reduce reliance on pyrethroids and enhance capacity to interrupt malaria transmission whilst living with pyrethroid resistance.

Organophosphates and carbamates, having a differing mode of action to pyrethroids, are potential alternative classes of insecticide, which could be used on wall linings [9]. These classes are effective against pyrethroid-resistant mosquitoes when used as IRS or wall linings [10, 11]. However, resistance to organophophates and carbamates due to insensitive acetylcholinesterase (*ace-1*^*R*^) has been reported in some pyrethroid-resistant malaria vector populations in West Africa [12–14]. Malaria vector control programmes, confronted by such multiple resistance, may be left with no option than resort to using these classes until new types of insecticide with novel modes of action are identified and made available.

The combining of non-pyrethroid IRS and pyrethroid LLIN has been recommended for resistance management and for improving control of insecticide-resistant malaria vectors [7]. This resistance management tactic relies on insect genotypes resistant to the insecticide in one intervention being killed by the insecticide in the other intervention provided they are not resistant to both insecticides [15]. Population genetics modelling indicates that combinations are less likely to provide this advantage when resistance to both insecticides is already present at detectable frequencies in the targeted vector population [8, 15]. However, reality is often more complex than the prediction of models. Some combinations may still improve personal protection or enhance kill through biochemical or behavioural interactions [7, 15]. With limited alternatives available for malaria control, empirical studies are needed to demonstrate whether improved vector control can be expected when non-pyrethroid IRS or wall linings are combined with pyrethroid LLINs against a multiple insecticide-resistant vector population.

In the current study, the efficacy of organophosphate-treated wall linings (WL) and net wall hangings (NWH) applied alone and in combination with LLINs was compared with pyrethroid-treated WL against an *Anopheles gambiae* population of Tiassalé, southern Côte d’Ivoire, which is resistant to the main classes of insecticide used in adult vector control [13]. Differential selection of insecticide-resistant genotypes was investigated to assess the potential for resistance management.

## Methods

### Susceptibility tests

The local *An. gambiae* mosquito population in Tiassalé has shown strong phenotypic resistance to the main classes of insecticides used for vector control: the resistance ratio was previously reported as 138-fold for the pyrethroid, deltamethrin and 24-fold for the carbamate, bendiocarb [13]. The Tiassalé population has the broadest resistance profile documented to date, with resistance being mediated by target site and metabolic mechanisms [13, 16, 17]. To assess the current levels of resistance to 0.05% deltamethrin and 0.25% p-methyl WHO susceptibility tests were performed on samples of adult *An. gambiae* that had emerged from larvae collected from the experimental hut site. A dosage of 0.25% was established as the diagnostic dosage for p-methyl using laboratory susceptible strains (H Ranson, pers comm).

### Experimental huts and study site

The trials were carried out in six experimental huts available in a rice field in Tiassalé (5°54′ N, 4°50′W), in southern Côte d’Ivoire. The rice paddies provide extensive breeding sites for mosquitoes throughout the year. The experimental huts were of the WHOPES-approved West African design [18, 19]. They were built on concrete plinths and surrounded by water-filled moats to prevent entry of scavenging ants. Veranda traps captured exiting mosquitoes. The huts were made of brick, plastered with cement, with corrugated iron roofs. The ceilings were made of high-density polyethylene sheeting and the walls had four window slits (with 1-cm gaps) through which mosquitoes could enter.

### Experimental hut treatments

Two experimental hut trials each lasting six weeks and involving six treatments were carried out against pyrethroid-resistant *An. gambiae* in Tiassalé. In the first trial, the efficacy of p-methyl-treated WL and NWH was evaluated, alongside the currently available deltamethrin WL (ZeroVector®, VestergaardFrandsen, Switzerland). Comparison of walls only and walls plus ceiling coverage were investigated:

1. Control (untreated plastic sheeting)
2. Pyrethroid (deltamethrin)-treated WL (ZeroVector®, Vestergaard Frandsen, Switzerland) on walls
3. P-methyl-treated WL on walls
4. P-methyl-treated NWH on walls
5. P-methyl WL on walls and ceilings
6. P-methyl NWH on walls and ceilings.

In the second hut trial, the p-methyl WL and NWH were combined with LLINs and compared to LLINs alone and p-methyl WL and NWH alone. The following six interventions were compared:

1. Untreated net with six holes
2. Pyrethroid LLIN (Permanet® 2.0 Vestergaard Frandsen, Switzerland), with six holes
3. P-methyl WL on walls and ceilings
4. P-methyl NWH on walls and ceilings
5. P-methyl WL on walls and ceilings + pyrethroid LLIN with six holes
6. P-methyl NWH on walls and ceilings + pyrethroid LLIN with six holes.

### Treatment of materials

The WL was 50% shade cloth made of woven high-density polyethylene (Capatex Ltd, UK). The NWH was made of 100-denier nylon netting fabric. These materials were treated at 1 g/sq m with a micro-encapsulated formulation of pirimiphos methyl (Actellic® 300CS Syngenta, Switzerland). The WL was treated by spraying with a Hudson Xpert sprayer, while the netting fabric was treated by hand dipping. Pyrethroid-treated WL was factory-made, high-density polyethylene fibre sheeting impregnated with deltamethrin at 175 mg/sq m (Zerovector®, Vestergaard Frandsen, Switzerland). The LLIN (PermaNet® 2.0, Vestergaard Frandsen, Switzerland) was WHOPES-approved, made of 100-denier polyester, factory-coated with a wash-resistant formulation of deltamethrin at a target dosage of 55 mg/sq m. To simulate wear and tear, the nets were intentionally holed with six 4-sq cm diameter holes (two on each side and one on each end) according to WHOPES guidelines [18]. The WL was fixed to the walls with nails while the NWH were hung from the top edge of the walls.

### Rotation of sleepers and treatments

Treatments were rotated weekly using a Latin square design to adjust for any differences in positional attractiveness of the huts. To prevent contamination between treatments during rotations, an underlay of untreated material was used to separate the treated materials from the walls and these were rotated with the treatments. The huts were also thoroughly washed before each rotation. Six adult men served as volunteer sleepers to attract mosquitoes into the huts, and were rotated between huts on successive nights to adjust for any variation in individual attractiveness to mosquitoes. The volunteers slept in the huts from 20:00 to 05:00 each night. Mosquitoes were collected each morning at 05:00 from under bed nets, floors, walls, ceilings, and verandas using aspirators and torches. The collections were identified to species and scored as blood fed or unfed and live or dead. Live mosquitoes were supplied with 10% glucose solution and delayed mortality was recorded after 24 hours.

### Main entomological outcomes

The entomological impact of each treatment in this study was expressed in terms of the following entomological outcomes:

1. Deterrence: percentage reduction in the number of mosquitoes caught in treated hut relative to the number caught in the control hut
2. Exiting rates: due to potential irritant effect of treatments expressed as percentage of the mosquitoes collected from the veranda trap
3. Inhibition of blood feeding: reduction in blood-feeding rate relative to the control:

where *Bfu* is the proportion of blood-fed mosquitoes in the untreated control huts and *Bft* is the proportion of blood-fed mosquitoes in the huts with a specific insecticide treatment

4. Mortality: percentage of dead mosquitoes in treated hut at the time of collection and after a 24-hour holding period corrected for control mortality
5. Personal protection: the proportional reduction in the number of blood-fed mosquitoes relative to blood-fed mosquitoes in the untreated control:

where *Bu* is the number of blood-fed mosquitoes in the untreated control huts and *Bt* is the number of blood-fed mosquitoes in the huts with a specific insecticide treatment.

### Residual activity of insecticide treatments

To measure residual activity, WHO cone bioassays were undertaken on treated materials *in situ* using the laboratory-susceptible *An. gambiae s.s.* Kisumu strain. Adult females three to five days old were exposed in cones fixed to plastic sheeting/NWHs for 30 minutes in accordance with WHO IRS guidelines [19]. Knockdown was recorded after one hour and mortality was recorded after 24 hours.

### Selection of insecticide resistance genes

Samples of *An. gambiae* collected from the respective experimental hut treatments through the course of the trials were preserved for molecular analysis. Genomic DNA was extracted using the Livak procedure [20]. Molecular detection of the *kdr* (L1014F) and *ace-1*^*R*^ (G119S) mutation alleles in live and dead samples from the hut treatments was carried out by real-time Taqman PCR as described by Bass *et al.* [21].

### Statistical analysis

The effects of each treatment on entomological outcomes (net penetration, blood-feeding, exiting, and mortality) were assessed using binomial generalized linear mixed models (GLMMs) with a logit link function fitted using the ‘lme4’ package of R version 2.12.2 for Windows [22]. A separate model was fitted for each outcome. In addition to the fixed effects, each model included random effects to account for variation between the six huts, between the six sleepers, between the six weeks of the trial, and finally an observation-level random effect was included to account for variation not explained by the other terms in the model (over-dispersion). Differences in deterrence, personal protection and exiting rates between the treatments was analysed using negative binomial regression with adjustment for variation between huts and sleepers, based on numbers entering, killed, and blood feeding, respectively.

Analysis of differential survival of genotypes for *ace-1*^*R*^ and *kdr* resistance by treatment was done using the Mantel-Haenszel Chi-squared test.

### Ethics statement

Ethical approval for the study was obtained from the Ethics Committee of the London School of Hygiene and Tropical Medicine (Approval No. 5872) and from the Ministry of Public Health of Côte d’Ivoire. Written informed consent was obtained from the sleeper volunteers.

## Results

### Susceptibility tests

The susceptibility tests confirmed that the *An. gambiae* population in Tiassalé were resistant to both deltamethrin and p-methyl. Mortality rates in WHO cylinder tests were 43% with deltamethrin 0.05% papers and 4% with p-methyl 0.25% papers (Table 1).

**Table 1:** **Susceptibility of wild** ***Anopheles gambiae*** **from Tiassalé to deltamethrin and p-methyl**

Species	Insecticide-treated papers	Number tested	24-hr% mortality (95% CI)
*An. gambiae* Tiassalés (wild resistant)	Deltamethrin 0.05%	99	43 (33–56)
	p-methyl 0.25%	99	4 (1–10)
*An. gambiae* Kisumu (susceptible lab strain)	Deltamethrin 0.05%	100	100 (96–100)
	p-methyl 0.25%	99	100 (96–100)

### Experimental hut trials

#### Single intervention trial

**Figure 1:**
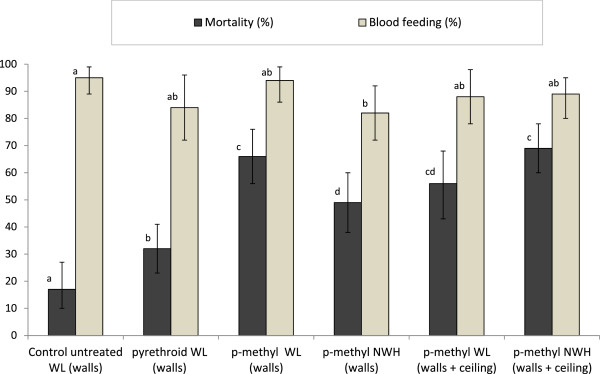
**Mortality and blood-feeding rates of multiple insecticide-resistant** ***Anopheles gambiae*** **(Tiassalé) in experimental huts (single interventions trial).** For each outcome (mortality or blood feeding), bars bearing the same letter label are not significantly different at the 5% level. Error bars represent 95% confidence intervals.

A total of 466 *An. gambiae* were collected in the six experimental huts during the single intervention trial. The results obtained are presented in Table 2 and Figure 1. As expected with such IRS-type treatments, overall blood-feeding rates were very high across all single WL and NWH treatments (range between treatments: 82 and 94%) and none of these differed significantly from the control (95%). Mortality rates were higher with p-methyl WL (66%) than with pyrethroid WL (32%) (Figure 1). The performance of p-methyl WL did not differ consistently from p-methyl NWH. Increasing the interior coverage with p-methyl WL and NWH from walls only to walls and ceilings showed, at best, only a small increase in mortality.

**Table 2:** **Efficacy of p-methyl wall lining and net wall hanging against pyrethroid-resistant** ***Anopheles gambiae*** **in Tiassalé, Côte d’Ivoire (single intervention trial)**

Hut treatment	Control (untreated WL)	Pyrethroid WL on walls	P-methyl WL on walls	P-methyl NWH on walls	P-methyl WL on walls and ceiling	P-methyl NWH on walls and ceiling
Total females caught	**53**	**114**	**98**	**70**	**54**	**77**
Deterrence (%)	-	0^a^	0^a^	0^a^	0^a^	0^a^
Total females blood fed	50	95	90	57	47	69
Blood-feeding inhibition (%)	-	12^a^	1^a^	14^a^	7^a^	6^a^
Personal protection (%)	0^a^	0^a^	0^a^	0^a^	6^a^	0^a^
Exiting rates (%)	45^a^	80^b^	36^a^	44^a^	31^a^	42^a^
Total dead	9	37	65	34	30	53
Corrected mortality	0^a^	18^b^	59^c^	39^d^	47^cd^	63^c^

Values along each row sharing the same letter superscript are not significantly different at the 5% level.

#### Combined intervention trial

**Figure 2:**
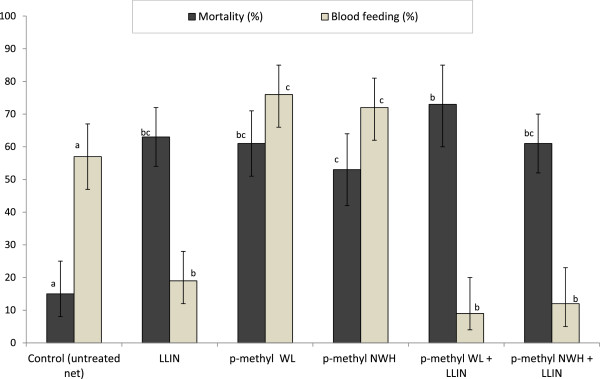
**Mortality and blood-feeding rates of multiple insecticide-resistant** ***Anopheles gambiae*** **(Tiassalé) in experimental huts (combined interventions trial).** For each outcome (mortality or blood feeding), bars bearing the same letter label are not significantly different at the 5% level. Error bars represent 95% confidence intervals.

A total of 557 *An. gambiae* were collected from the experimental huts during the combination trial. The results are presented in Table 3 and Figure 2. Blood feeding with the LLIN was significantly lower than with the untreated net (19 *vs* 57%; P < 0.001). Blood-feeding rates were higher with the p-methyl WL (76%) and NWH treatments (72%) when applied alone. When p-methyl WL and NWH were combined with LLINs, blood-feeding rates reduced significantly to 9 and 13%, respectively; these rates were not significantly different from those with the LLIN treatment. Thus the lower feeding rates associated with the combinations can be attributed to the LLIN component. The combination treatments conferred significantly more personal protection than the p-methyl WL or NWH alone (93 *vs* 0% and 92 *vs* 4%, respectively) (P < 0.001) (Table 3).

**Table 3:** **Efficacy p-methyl wall lining and net wall hanging combined with long-lasting insecticidal nets against multiple insecticide-resistant** ***Anopheles gambiae*** **in Tiassalé, Côte d’Ivoire (combined intervention trial)**

Hut treatment	Control (untreated net)	LLIN	P-methyl WL	P-methyl NWH	P-methyl WL + LLIN	P-methyl NWH + LLIN
Total females caught	**130**	**108**	**94**	**126**	**53**	**46**
Deterrence (%)	-	17^a^	28^a^	3^a^	59^b^	65^b^
Total females blood fed	74	20	71	91	5	6
Blood-feeding inhibition (%)	-	67^a^	0^b^	0^b^	84^a^	77^a^
Personal protection (%)	0^a^	73^b^	4^c^	0^c^	93^b^	92^b^
Total inside net (%)	54^a^	15^b^	-	-	6^b^	10^b^
Exiting rates (%)	29 ^a^	51^bc^	53^b^	38^ac^	33^a^	59^b^
Total dead	20	68	57	67	38	28
Corrected mortality (%)	0^a^	56^bc^	54^bc^	45^b^	67^c^	54^bc^

Values along each row sharing the same letter superscript are not significantly different at the 5% level.

Mortality of *An. gambiae* with the LLIN (63%) was significantly higher than with the untreated net (15%) (P < 0.001) but did not differ significantly from the p-methyl WL alone (63 *vs* 61%; P = 0.68) or p-methyl NWH alone (63 *vs* 53%; P = 0.07). Mortality rates with the combinations were 72% for p-methyl WL plus LLIN and 61% for p-methyl NWH plus LLIN and neither of these values differed significantly from the LLIN alone (P > 0.05), p-methyl WL (72 *vs* 61%, P = 0.06) or NWH single treatments (61 *vs* 53%, P = 0.78) (Figure 2). Thus the two combination treatments failed to induce significantly higher levels of mortality than the respective single treatments.

### Resistance selection studies with *Anopheles gambiae s.s.*

The overall *kdr* and *ace-1*^*R*^ allele frequencies were, respectively, 0.83 and 0.44 during the trials. Tables 4 and 5 present the allele and genotype frequencies for *kdr* and *ace-1*^*R*^*.* The frequency of the *kdr* allele did not differ between the live and dead collections of any of the treatments during either the single (M-H Chi sq = 0.2, P = 0.65) or combined intervention trial (M-H Chi sq = 1.6, P = 0.21) (Table 4). The *ace-1*^*R*^ allele frequency during the single intervention trial was generally higher in the live collections of the p-methyl WL and NWH interventions than in the dead collections (M-H Chi square = 12.9, df = 1, P = 0.0003); this indicates that *ace-1*^*R*^ bearing mosquitoes were more likely to survive in huts with p-methyl treatments. However, during the combined intervention trial, the *ace-1*^*R*^ allele frequencies in the single p-methyl interventions did not differ significantly between the live and dead collections (M-H Chi sq = 1.8, P = 0.18); in the combination interventions the *ace-1*^*R*^ allele frequency was actually higher in the live than in the dead collections but numbers collected were low and the difference in frequency between the live and dead samples was not significant (M-H Chi sq = 1.0, P = 0.32).

**Table 4:** **Comparative** ***kdr*** **and** ***ace-1*** ^***R***^ **allele frequencies in live and dead** ***Anopheles gambiae*** **collected from experimental huts in Tiassalé**

		***kdr***allele frequency (n)		***ace-1*** ^***R***^allele frequency (n)	
Treatments		Live	Dead	Live	Dead
**First trial (single intervention)**					
**1**	Pyrethroid WL	0.87 (75)	0.89 (38)	--	--
**2**	P-methyl WL (walls only)	0.81 (16)	0.83 (32)	0.44 (25)	0.38 (73)
**3**	P-methyl NWH (walls only)	0.81 (16)	0.88 (16)	0.45 (33)	0.43 (34)
**4**	P-methyl WL (walls and ceiling)	0.91 (16)	0.89 (22)	0.46 (24)	0.33 (32)
**5**	P-methyl NWH (walls and ceiling)	0.75 (12)	0.83 (20)	0.52 (25)	0.44 (45)
**Second trial (combined intervention)**					
**1**	Control (untreated net)	0.91 (76)	0.91 (16)	0.48 (77)	0.40 (15)
**2**	LLIN	0.88 (41)	0.86 (62)	--	--
**3**	P-methyl WL	0.88 (24)	0.79 (24)	0.52 (24)	0.50 (24)
**4**	P-methyl NWH	0.79 (24)	0.83 (20)	0.48 (24)	0.45 (21)
**5**	P-methyl WL + LLIN	0.95 (11)	0.89 (41)	0.50 (11)	0.43 (42)
**6**	P-methyl NWH + LLIN	0.84 (19)	0.81 (18)	0.55 (19)	0.39 (18)

**Table 5:** **Genotype selection by the single and combination treatments: percentage survival of** ***Anopheles gambiae kdr*** **and** ***ace-1*** ^***R***^ **genotypes collected from the experimental huts in Tiassalé**

		***kdr***: % alive (live/total)			***ace 1*** ^***R***^: % alive (live/total)		
Treatments		***SS***	***RS***	***RR***	***SS***	***RS***	***RR***
**First trial (single intervention)**							
**1**	Pyrethroid WL	33 (1/3)	73 (16/22)	65 (57/88)	-	-	-
**2**	P-methyl WL (walls only)	0 (0/3)	55 (6/11)	29 (10/34)	15 (3/20)	28 (22/78)	-
**3**	P-methyl NWH (walls only)	75 (3/4)	0 (0/2)	50 (13/26)	25 (3/12)	59 (30/51)	0 (0/4)
**4**	P-methyl WL (walls and ceiling)	0 (0/2)	75 (3/4)	41 (13/32)	15 (2/13)	51 (22/43)	-
**5**	P-methyl NWH (walls and ceiling)	0 (0/1)	55 (6/11)	30 (6/20)	0 (0/9)	43 (24/56)	25 (1/4)
**Second trial (combined intervention)**							
**1**	Control (untreated net)	100 (2/2)	77 (10/13)	83 (64/77)	70 (7/10)	85 (66/78)	100 (4/4)
**2**	LLIN	0 (0/2)	43 (10/23)	40 (31/78)	-	-	-
**3**	P-methyl WL	0 (0/2)	50 (6/12)	53 (18/34)	17 (1/6)	70 (23/33)	40 (2/5)
**4**	P-methyl NWH	0 (0/1)	67 (10/15)	50 (14/28)	43 (3/7)	56 (19/34)	50 (2/4)
**5**	P-methyl WL + LLIN	0 (0/2)	20 (1/5)	23 (10/44)	20 (2/10)	18 (7/39)	50 (2/4)
**6**	P-methyl NWH + LLIN	0 (0/2)	67 (6/9)	50 (13/26)	17 (1/6)	56 (15/27)	75 (3/4)

SS = susceptible homozygotes, RS = resistant heterozygotes, RR = resistant homozygotes.

Analysis by genotype reveals further trends (Table 5). There were very few mosquitoes bearing no *kdr* allele. There was no significant difference in the percentage survival of homozygotes for *kdr* (40%) over heterozygotes for *kdr* (43%) during exposure to the LLIN treatment despite *kdr* resistance being supposedly recessive. The addition of p-methyl NWH or WL to the LLIN in the combination interventions did not affect the survival of heterozygotes for *kdr* relative to homozygotes for *kdr* but did increase the proportions of these genotypes killed. With respect to the *ace-1*^*R*^, heterozygotes (*RS*) and homozygotes for *ace-1*^*R*^*(RR)* showed higher percentage survival than susceptible homozygotes *(SS)* on exposure to p-methyl WL or NWH single treatments, both in the first trial (M-H Chi sq = 16.6, P < 0.001) and in the second (M-H Chi sq = 5.1, P = 0.02). This indicated selection for *ace-1*^*R*^ and shows the importance of analysis by genotype. With the combination intervention of LLIN and p-methyl NWH the trend remained in this direction, with selection of *ace-1*^*R*^ genotypes. With the combination of LLIN and p-methyl WL there was, on this occasion, no trend that favoured survival of *ace-1*^*R*^ genotypes (*RR* and *RS*) over susceptible homozygotes (*SS*). Overall there was no clear evidence to indicate that the addition of LLIN to p-methyl-treated WL or NWH would prevent the selection of *ace-1*^*R*^ homozygotes and heterozygotes (*RR* and *RS*) relative to the susceptible homozygotes (*SS*). All three genotypes showed quite high levels of survival against single p-methyl and combination interventions. There were many more resistant heterozygotes (*RS*) and far fewer resistant homozygotes (*RR)* collected than would be expected from Hardy-Weinberg ratios.

### Residual efficacy

The residual efficacy of the p-methyl WL and NWH as determined by cone bioassays using *An. gambiae* Kisumu declined from 100% during the first two to three weeks of the trial to 60-70% by the end of the trial.

## Discussion

The aim of the study was to evaluate the efficacy and selection of resistance by p-methyl-treated WL when either applied alone or in combination with LLINs against an *An. gambiae* population in southern Côte d’Ivoire, which was resistant to pyrethroids and organophosphates [13]. The reported trial was part of a multicentre trial designed to demonstrate as a proof of concept whether non-pyrethroid wall liners could provide benefits for control when combined with LLINs against malaria vector populations with differing levels of insecticide resistance. It was also hoped to assess their potential for resistance management. In the trial of similar interventions conducted in Burkina Faso where vectors were also resistant to pyrethroids but largely susceptible to organophosphates, the p-methyl WL and NWH were far more effective, killing almost all mosquitoes (>95%) that entered the huts even without the addition of LLIN [23]. The lower mortality rates achieved with p-methyl-treated WL and NWH in the Côte d’Ivoire study (50-65%) can therefore be attributed to the high levels of phenotypic resistance to organophosphates. Despite the poorer levels of control relative to the Burkina Faso study, p-methyl WL and NWH, proved to be a better option against this multiple insecticide-resistant vector population than commercial pyrethroid WL, which killed only 30% of mosquitoes entering the huts.

High vector mortality and personal protection against biting mosquitoes are the desired outcomes of any vector control tool or combination of tools. LLINs are very efficacious in areas of full susceptibility to pyrethroids, where they can induce high mortality rates in mosquito populations and provide personal protection to net users [24]. Although the insecticidal efficacy of LLINs may be compromised when confronted with moderate to high pyrethroid resistance, LLINs can still be protective as shown in both the present Côte d’Ivoire and previous Burkina Faso studies [23] owing to the barrier effect of the net and the residual killing effect of the pyrethroid. Hence, LLINs can remain an important public health intervention even against malaria vector populations, which have moderate levels of resistance to pyrethroids [8]. Against vector populations which are resistant to pyrethroids but largely susceptible to the insecticide applied on the walls, the combining of pyrethroid LLINs with non-pyrethroid IRS has shown, in small scale hut trials, improved levels of mortality (mostly due to the wall treatment) and improved personal protection (due to the LLIN) [10, 25]. Under such circumstances the combination appears to restore mortality rates to levels comparable to that achieved with LLINs alone in areas where vectors are susceptible to pyrethroids [24–26]. In the present study, the combination failed to provide improved mortality over the LLIN alone against a multiple insecticide-resistant malaria vector population. This is a very disturbing finding considering the limited classes of insecticides currently available for malaria vector control. Until a class of insecticide with a novel mode of action is developed for vector control, malaria programmes faced with such multiple insecticide resistance may have no suitable alternatives to complement or provide a boost to failing LLINs. The study demonstrates the threats and challenges that the malaria vector control community will face if such resistance to multiple insecticides is left unchecked and continues to spread.

In other parts of West Africa, the *ace-1*^*R*^ gene has often been reported in pyrethroid-resistant *An. gambiae* populations at low frequencies [12, 23, 27, 28]. While heterozygotes for *ace-1*^*R*^ did show some selective advantage over homozygotes for susceptibility in the Burkina Faso study [23], the Côte d’Ivoire Tiassalé population had a far higher frequency of *ace-1*^*R*^ and the use of organophosphate WL clearly demonstrated the survival and selection of *ace-1*^*R*^ genotypes. A parallel mechanistic investigation on the Tiassalé population has demonstrated gene duplication at the *ace-1*^*R*^ locus [16]; the duplication may account for the dominance and survival advantage of *ace-1*^*R*^ genotypes and would also explain the departure from Hardy-Weinberg expectation and the surplus of heterozygotes. While the number of mosquitoes collected and analysed for *ace-1*^*R*^ in the second (combination) trial was not huge, there was no convincing evidence that *ace-1*^*R*^ heterozygotes or homozygotes were less likely to survive exposure to the combination relative to the single p-methyl interventions or that the combination would manage resistance. This, together with the quite high survival rates among mosquitoes that bore *no ace-1*^*R*^ alleles, suggests the presence of another mechanism, independent of *ace-1*^*R*^, going undetected in survivors, which was partly responsible for organophosphate resistance. Recent studies showed improved mortality of *An. gambiae* from Tiassalé exposed to bendiocarb, pyrethroids and an organophosphates (fenitrothion) with different synergists, thus implicating enhanced P450s and esterases in the resistance to all three classes of insecticide [16, 17, 29]. An investigation of the genetic basis of resistance in the Tiassalé population has associated genes from the CYP6 subfamily with resistance to pyrethroids and carbamates [16]. It is important to identify the specific enzyme families, which in association with the *ace-1*^*R*^ mechanism, combine to increase resistance to p-methyl in this vector population.

While no large-scale community trial has been published on the combined effects of pyrethroid LLIN and organophosphate IRS compared to LLIN alone, two community randomized trials of LLIN and carbamate IRS have been published recently: one in Tanzania [30] and one in Benin [31]. Both were in areas of high-frequency pyrethroid resistance and low-frequency carbamate resistance. The Tanzanian trial showed an added effect of the combination over LLIN alone, and this result was therefore consistent with the outcome of the Burkina Faso experimental hut trial of LLIN and OP wall liners (and local susceptibility status). The contrasting findings from the two multicentre hut trials in Burkina Faso and Côte d’Ivoire illustrate the uncertainty of outcome when faced with resistance to multiple insecticides rather than single insecticides. From the outcome of the Côte d’Ivoire trial, there can be no doubt that selection of multiple insecticide resistance will only make it harder to control malaria.

## Conclusion

P-methyl WL and NWH performed better than pyrethroid WL against multiple pyrethroid and organophosphate-resistant *An. gambiae*. Combining p-methyl WL and NWH with LLINs provided no improvement in mortality and personal protection compared to the LLIN alone. There was no evidence that the combination of pyrethroid LLIN and organophosphate WL would prevent the selection of either *kdr* or *ace-1*^*R*^ resistance when both are present at detectable or moderate frequencies. The study demonstrates the challenge that malaria vector control programmes are faced with when confronted with such high levels of phenotypic resistance to multiple insecticides. Strategies of insecticide deployment or rotation to delay the rapid spread of the *ace-1*^*R*^ gene in Africa and the further development of multiple insecticide-resistant vector populations are urgently required.
